# Clinical Effectiveness and Safety of Once-Weekly GLP-1 Receptor Agonist Dulaglutide as Add-On to Metformin or Metformin Plus Insulin Secretagogues in Obesity and Type 2 Diabetes

**DOI:** 10.3390/jcm10050985

**Published:** 2021-03-02

**Authors:** Maria Mirabelli, Eusebio Chiefari, Vera Tocci, Patrizia Caroleo, Stefania Giuliano, Emanuela Greco, Raul Miguel Luque, Luigi Puccio, Daniela Patrizia Foti, Antonio Aversa, Antonio Brunetti

**Affiliations:** 1 Department of Health Sciences, University “Magna Græcia” of Catanzaro, 88100 Catanzaro, Italy; maria.mirabelli@unicz.it (M.M.); echiefari@gmail.com (E.C.); emanuela.greco@unicz.it (E.G.); foti@unicz.it (D.P.F.); 2 Unit of Endocrinology, Azienda Ospedaliera Mater-Domini, 88100 Catanzaro, Italy; tocci.vera@gmail.com (V.T.); stefania.giuliano75@gmail.com (S.G.); aversa@unicz.it (A.A.); 3 Unit of Endocrinology and Diabetes, Azienda Ospedaliera Pugliese-Ciaccio, 88100 Catanzaro, Italy; patrizia.caroleo@alice.it (P.C.); puccio55@libero.it (L.P.); 4 Department of Cell Biology, Physiology and Immunology, University of Córdoba, 14071 Córdoba, Spain; bc2luhur@uco.es; 5 Department of Clinical and Experimental Medicine, University “Magna Græcia” of Catanzaro, 88100 Catanzaro, Italy

**Keywords:** GLP-1 receptor agonist (GLP-1 RA), dulaglutide, liraglutide, add-on therapy, type 2 diabetes, obesity

## Abstract

Aims and methods: The aim of this monocentric retrospective observational study was to evaluate the 18-month safety and effectiveness of GLP-1 receptor agonist (GLP-1 RA) dulaglutide (DU) 1.5 mg/once weekly as an add-on to metformin (MET) or MET plus conventional insulin secretagogues in a study cohort with excess body weight and type 2 diabetes (T2D). Comparative efficacy versus liraglutide (LIRA) 1.2–1.8 mg/once daily in a study sample naïve to GLP-1 RAs, frequency matching for age, gender, T2D duration, degree of glycemic impairment, cardiovascular comorbidities, and medications, was addressed as a secondary aim. Clinical and biochemical data for efficacy outcomes and information on drug discontinuation due to adverse events (AEs) were collected from digital records. Results: Initial analysis included 126 overweight and obese T2D patients (48.4% females). Out of these, 13 discontinued DU due to moderate–severe gastrointestinal AEs after a mean follow-up of 6 (4 standard deviations (SD)) months, while 65 completed 18 months of continuous therapy. At 6 months, there was a significant mean HbA1c reduction of −0.85% (1.17 SD) with respect to baseline values (*p* < 0.001), which remained stable during 18 months follow-up. These results were accompanied by a moderate weight loss sustained over time, with a mean reduction of −2.0% (4.3 SD) at 6 months and −1.3% (4.8 SD) at 18 months (*p* = 0.091). At univariate analysis, a negative correlation between baseline body mass index (BMI) and risk of drug discontinuation due to gastrointestinal AEs was observed. The protective effect of obesity against drug discontinuation was confirmed by logistic regression analysis. Neither gender, nor age, nor T2D duration, nor concomitant conventional insulin secretagogue use, nor switching to DU from other GLP-1 RAs influenced its long-term effectiveness. However, higher baseline HbA1c values emerged as predictors of clinically relevant efficacy outcomes, either in terms of HbA1c reduction ≥ 0.5% or body weight loss ≥ 5%. The efficacy outcomes were corroborated by head-to-head comparison with LIRA, a GLP-1 RA with durable beneficial effects on glycemic control and body weight in real-world experiences. With the advantage of once-weekly administration, at 18-month follow-up, a significantly larger fraction of patients on DU therapy reached glycemic targets (HbA1c ≤ 7.0%) when compared to those on LIRA: from 14.8% at baseline (both groups) to 64.8% with DU and 42.6% with LIRA (*p* = 0.033). Conclusions: Although limited by a retrospective design and lack of constant up-titration for LIRA to the highest dose, these findings indicate that the beneficial responses to DU on a background of MET or MET plus insulin secretagogues are durable, especially in the presence of obesity and greater HbA1c impairment.

## 1. Introduction

Leading consensus recommendations recognize that metformin (MET) should be the first-line drug therapy choice for the management of hyperglycemia in type 2 diabetes (T2D) [1]. If MET on its own does not suffice to control glucose, a second-line drug may need to be added. Due to low costs, wide availability, oral administration, and evidence of benefits comparable to those of other glucose-lowering agents on most patient-important outcomes (i.e., mortality, microvascular complications) [2], the conventional insulin secretagogues, such as sulfonylureas and glinides, remain some of the most commonly prescribed medications, either alone or on top of MET [2,3]. Despite some advantages, induction of hyperinsulinemia by these drugs can increase the risk of hypoglycemic adverse events (AEs) and body weight gain, posing considerable problems in the long run [2,4]. Preferable options in patients with inadequately controlled T2D and excess body weight are glucagon-like peptide-1 receptor agonists (GLP-1 RAs), such as dulaglutide (DU) and liraglutide (LIRA), both delivered by subcutaneous injections [1,4]. Contrary to sulfonylureas and glinides, GLP-1 RAs stimulate insulin secretion in a glucose-dependent manner, minimizing the risk of hypoglycemia. In addition, GLP-1 RAs have been shown to promote satiety signals and delay gastric emptying, thus reducing postprandial glycemic and insulinemic peaks, while favoring weight loss [5]. Multiple lines of evidence indicate that losing as little as 5% of the initial body weight may improve insulin resistance in individuals with obesity [6], as well as glycemic control and other cardiometabolic markers in overweight and obese patients with T2D, thereby reducing the risk of acute cardiovascular events, the leading cause of morbidity and mortality [4,7]. Conversely, drug-induced gain in body weight may retain the potential to offset the beneficial effects of glycemic control on cardiovascular risk [8,9].

GLP-1 RAs differ with respect to molecular structure and pharmacokinetic properties, including absorption and clearance, so that certain molecules (i.e., DU, exenatide LAR) are suitable for once-weekly injections, whereas others (i.e., LIRA, lixisenatide) need to be administered once daily, or even more frequently, because of a shorter half-life [10]. Steady-state plasma DU concentrations are achieved between 2 and 4 weeks following once-weekly administration, and no dose adjustments are required in case of liver impairment or mild–moderate kidney failure. As a second-line, adjunctive therapy, the recommended DU dose to improve glycemic control is 1.5 mg/once weekly [11]. Efficacy and safety of DU as add-on therapy to MET or a combination of MET plus a sulfonylurea in patients with inadequately controlled T2D have been, respectively, demonstrated in the open-label phase-III clinical trials AWARD-5 [12] and AWARD-2 [13]. In both clinical experimental conditions, DU maintained a significant advantage over placebo and active comparators, such as sitagliptin and insulin, for up to 18 months [12,13]. Less is known about the comparative efficacy of DU with respect to other GLP-1 RAs from head-to-head clinical trials [14]. More recently, many post-marketing observational studies, addressing either the clinical effectiveness and safety of DU or the predictors of a better glycometabolic response under routine endocrinology practice conditions, have been conducted in European and Asian countries [15,16,17,18,19,20], in which, however, heterogeneous results were found. This was probably due to the short duration of follow-up and the differences in background antidiabetic medications at study entry. Furthermore, none of the studies included patients from Calabria, a Southern Italian region with a highly genetically homogeneous population structure that constantly keeps negative records for the prevalence of T2D and excess body weight [21,22]. Thus, the aim of the present study was to evaluate the 18-month safety and effectiveness of DU 1.5 mg/once weekly as add-on therapy to MET or a combination of MET plus conventional insulin secretagogues in a patient cohort with T2D and overweight/obesity attending our endocrinology outpatient clinic. Comparative efficacy versus LIRA 1.2–1.8 mg/once daily, a GLP-1 RA with durable beneficial effects on glycemic control and body weight management in the Calabrian population [9,22], was addressed as a secondary aim.

## 2. Materials and Methods

### 2.1. Study Participants 

**Figure 1:**
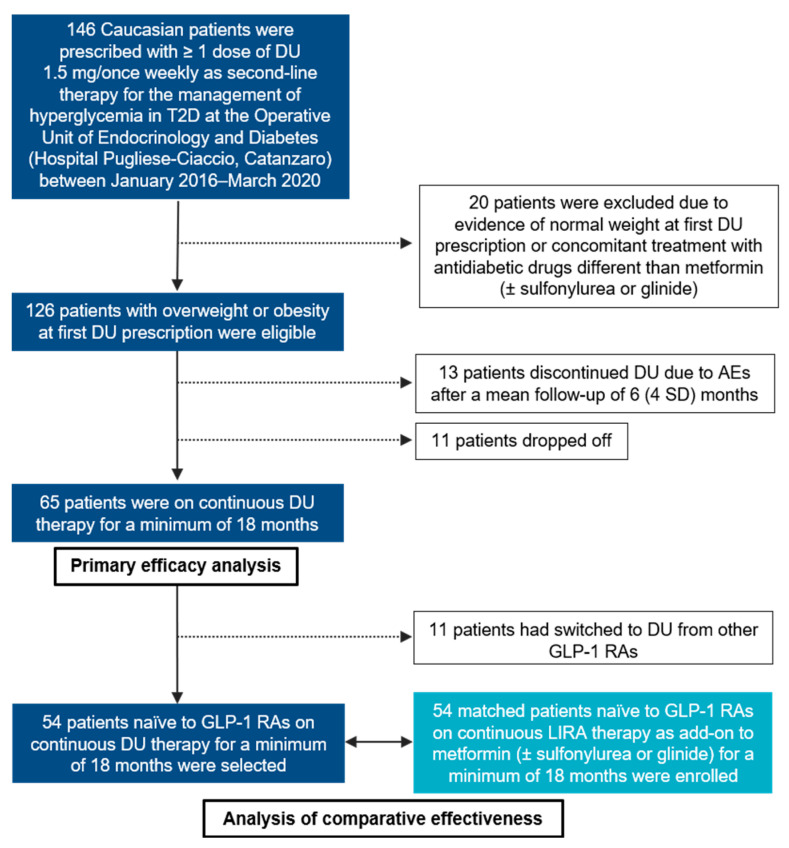
Flowchart of the study. DU: dulaglutide; GLP-1 RA: GLP-1 receptor agonist; T2D: type 2 diabetes; AEs: adverse events; SD: standard deviations.

In this monocentric, retrospective, observational cohort study, we analyzed the 18-month safety and effectiveness of DU 1.5 mg/once weekly (*Trulicity*^®^, Eli Lilly, Indianapolis, IN, USA) as a second-line, adjunctive therapy to MET or a combination of MET plus conventional insulin secretagogues (sulfonylurea, glinide) in a Southern Italian population of overweight (body mass index, BMI 25–29.9 kg/m^2^) and obese (BMI ≥ 30 kg/m^2^) Caucasian subjects affected by T2D. Consecutive patients with at least one prescription of DU during the period January 2016–March 2020 were recruited from the operative Unit of Endocrinology and Diabetes at Hospital “Pugliese-Ciaccio” in Catanzaro. Normal weight (BMI ≤ 25 kg/m^2^) participants at the first DU prescription, or participants concomitantly treated with other glucose-lowering drugs, except for MET and insulin secretagogues (i.e., insulin), were excluded from the primary analysis. Age, gender, T2D duration, body weight, BMI, blood pressure (BP), lipid profile, fasting plasma glucose (FPG), HbA1c, aspartate aminotransferase/alanine aminotransferase (AST/ALT), serum creatinine with estimated glomerular filtration rate (eGFR), micro- and macrovascular complications, and any concomitant pharmacological therapy were recorded at baseline for all patients. An additional study cohort, naïve to GLP-1 RAs and on continuous therapy with LIRA 1.2–1.8 mg/once daily (*Victoza*^®^, Novo Nordisk, Bagsværd, Denmark) for a minimum of 18 months, frequency-matching for age, gender, T2D duration, degree of glycemic impairment, cardiovascular comorbidities, and background medications, were enrolled for the secondary aim (Figure 1).

### 2.2. Assessments

Following the prescription of DU 1.5 mg/once weekly as second-line therapy for the management of hyperglycemia in T2D, according to the Italian scientific diabetes societies (AMD-SID) consensus recommendations [23] and national reimbursement criteria, all patients underwent periodical clinical and biochemical evaluations at 6-month intervals to monitor safety and efficacy of the drug. The variables analyzed to assess safety included: creatinine-based eGFR, AST/ALT liver enzymes, and the occurrence of drug discontinuation due to AEs. The variables analyzed to assess efficacy included: body weight, BMI, HbA1c, FPG, systolic and diastolic BP. Any serious medical problems reported during follow-up, including incident cardiovascular events, were recorded on an electronic patient diary (Smart Digital Clinic^®^, Meteda Srl, San Benedetto del Tronto, Italy) and the entries were reviewed at each study visit.

### 2.3. Outcome Measures

The primary outcome was to test the 18-month safety of DU 1.5 mg/once weekly under routine conditions of use. Safety outcome measures were the longitudinal changes in AST/ALT liver enzymes, eGFR, and the proportion of participants stopping DU therapy due to AEs during follow-up. The secondary outcome was to test the clinical effectiveness of DU treatment for glycemic control and body weight management. Efficacy outcome measures were the longitudinal changes from baseline in HbA1c and body weight for up to 18 months of continuous therapy, and the proportions of participants with either relative HbA1c reduction ≥ 0.5% or body weight loss ≥ 5% at the end of the study period. Other efficacy outcome measures included longitudinal changes in FPG, BP, lipid profile, and proportions of participants achieving glycemic targets recommended by the American Diabetes Association (ADA) “Standards of Medical Care in Diabetes” guidelines (HbA1c level ≤ 7.0%) [1]. Finally, we searched for potential baseline predictors of better responses to DU and corroborated the efficacy outcomes by head-to-head comparison with LIRA.

### 2.4. Statistical Analysis

Continuous traits were expressed as means and standard deviations (SD), while categorical traits as numbers and percentages. The non-parametric Wilcoxon signed-rank test was used for longitudinal within-group comparisons, whereas the Mann–Whitney U test was employed to determine the presence of significant differences in head-to-head comparisons between frequency-matched DU- and LIRA-treated participants. The 2-tailed Fisher exact test was used to compare proportions. The Spearman’s rank correlation analysis was performed to identify any predictive factor of either risk of drug discontinuation due to AEs or the occurrence of clinically relevant efficacy outcomes for the DU-treated participants. Then, significant variables were forced into logistic regression models with appropriate covariate adjustment, and odds ratios (OR) with 95% confidence intervals as relative effect estimates were calculated. A significance level of 0.05 was set for all analyses. Data were analyzed with JASP Graphical Statistical Software Version 0.14.1 (University of Amsterdam, Amsterdam, The Netherlands) based on R Stats packages.

## 3. Results

### 3.1. Baseline Characteristics of the Study Cohort

Out of 146 patients with at least one prescription of DU 1.5 mg/once weekly during the study period, a total of 126 were eligible for enrollment and initial analysis (Figure 1). As reported in Table 1, 48.4% of study participants were women, the mean age was 59.8 (9.4 SD) years and the mean T2D duration was 8.0 (6.0 SD) years. Given the short duration of the disease, only a minority of patients (18.3%) suffered from microvascular diabetic complications, such as diabetic retinopathy, diabetic kidney disease (DKD), diabetic neuropathy, or a combination of any of them (Table 1). According to pre-specified BMI categories, 46 (36.5%) patients in this study were classified as overweight, whereas 80 (63.5%) had obesity. When initiating DU treatment as adjunctive therapy to MET or MET plus insulin secretagogues (sulfonylurea, 14.2%; glinide, 7.1%), 17 (13.5%) patients were switching from other currently used GLP-1 RAs, namely LIRA and lixisenatide, both dosed once daily [10] (Table 1). 

### 3.2. Safety Outcomes

Out of 126 eligible participants, 13 (10.3%) discontinued DU due to moderate–severe gastrointestinal AEs (i.e., nausea, vomiting, and diarrhea, requiring in one case a short hospital stay because of severe dehydration) after a mean follow-up of 6 (4 SD) months (Figure 1). None of the patients included in the primary analysis discontinued DU due to severe hypoglycemic events (i.e., requiring the assistance of another individual), acute pancreatitis or injection site reactions, whereas two patients experienced a first non-fatal, acute cardiovascular event (one myocardial infarction and one cerebral ischemia) and one patient was admitted to the endocrinology unit for very poor glycemic control. Notwithstanding uncertainties surrounding the estimates due to limited data, a slight but significant reduction in mean creatinine-based eGFR was observed at 18-month follow-up, from 90.1 (25.1 SD) at baseline to 84.1 (20.7 SD) mL/min (*p* = 0.049), without clinically meaningful kidney-related AEs (i.e., rising of serum creatinine over 30%), causing drug discontinuation [21,24]. No significant variations were detected for the AST/ALT liver enzymes (Table 2). 

### 3.3. Efficacy Outcomes and Predictors of Response

**Figure 2:**
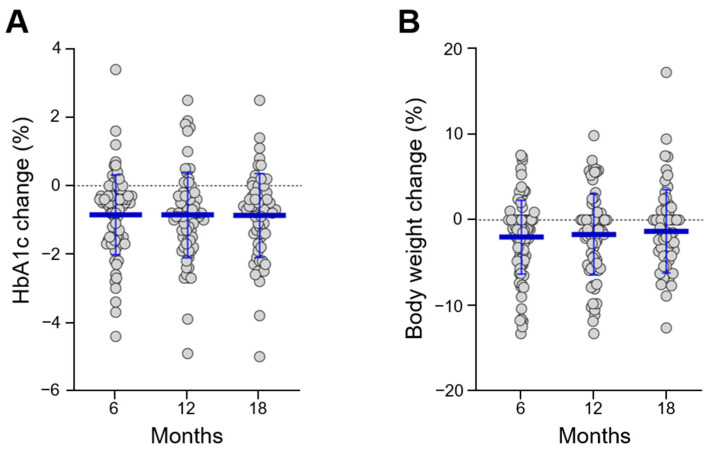
Longitudinal changes in HbA1c (**A**) and body weight (**B**) for up to 18 months. Longitudinal changes were calculated with respect to baseline values, as absolute (HbA1c) or relative (body weight) percentage differences.

As shown in Figure 1, we were able to follow 65 (51.6%) participants on continuous DU therapy for up to 18 months. At 6-month follow-up, there was a significant mean HbA1c reduction of −0.85% (1.17 SD) with respect to baseline values (*p* < 0.001), which remained stable until the end of the study (Supplementary Table S1). These results were accompanied by a moderate weight loss sustained over time, with a mean reduction of −2.0% (4.3 SD) of initial body weight at 6 months and −1.3% (4.8 SD) at 18 months (*p* = 0.091) (Supplementary Table S1). Figure 2 provides a graphical representation of the longitudinal changes in HbA1c and body weight observed in our cohort with jitter points and error bars.

Table 3 shows the longitudinal comparisons of secondary efficacy outcomes measures at 6-month intervals for up to 18 months. As expected from the beneficial effects of DU on lipid metabolism consistently reported in clinical trials [12,13,25], a significant improvement in total cholesterol, low-density lipoprotein (LDL)-cholesterol, and triglycerides could be demonstrated at all study visits, without intensification of any background lipid-lowering therapy (Table 3). No significant differences emerged on HDL-cholesterol levels and BP.

Searching for predictors of better responses to DU, by univariate Spearman’s rank correlation analysis, we observed a negative correlation between baseline BMI and risk of drug discontinuation due to gastrointestinal AEs (ρ = −0.190, *p* = 0.035). The protective effect of obesity against drug discontinuation was confirmed by logistic regression analysis while adjusting for appropriate covariates (OR 0.211 (95% confidence intervals (CI) 0.058–0.771), *p* = 0.019). Neither gender, nor age, nor T2D duration, nor concomitant conventional insulin secretagogue use, nor switching to DU from other GLP-1 RAs influenced its long-term effectiveness. However, higher baseline HbA1c values were predictive of clinically relevant efficacy outcomes, either in form of HbA1c reduction ≥ 0.5% (OR 2.961 (95% CI 1.394–6.290), *p* = 0.005) or body weight loss ≥ 5% (OR 2.571 (95% CI 1.171–5.644), *p* = 0.019) (Table 4). 

### 3.4. Comparative Efficacy versus Liraglutide

Finally, we corroborated the efficacy outcomes by head-to-head comparison with LIRA, a GLP-1 RA with long-lasting beneficial effects on glycemic control and body weight management, as demonstrated by real-word experiences from our group and others [9,26]. For this aim, two matched groups of 54 participants, naïve to GLP-1 RAs, who were prescribed DU 1.5 mg/once weekly or LIRA 1.2–1.8 mg/once daily as add-on therapy to MET or a combination of MET plus conventional insulin secretagogues, for a minimum of 18 months, were selected, controlling for age, gender, T2D duration, degree of glycemic impairment, cardiovascular comorbidities, and background medications. Frequency matching into data collection allowed to balance the potential confounding factors when addressing real-world efficacy outcomes of GLP-1 RAs in cohort studies with an active comparator design [27], reducing the need for further stratification in statistical analysis [28]. Supplementary Table S2 shows that treatment groups were highly comparable with respect to baseline characteristics. With the advantage of once-weekly administration, at 18-month follow-up, a significantly larger fraction of patients on DU therapy reached the recommended glycemic targets (HbA1c ≤ 7.0%) when compared to those on LIRA: from 14.8% at baseline (both groups) to 64.8% with DU and 42.6% with LIRA (*p* = 0.033) (Table 5). However, similar to other real-world experiences [9,26,29], only in 54% of participants LIRA was up-titrated to the highest dose. Supplementary Table S3 shows the comparisons of longitudinal data at 6 months intervals between matched groups.

## 4. Discussion

DU 1.5 mg/once weekly has been compared to insulin when treatment with a combination of MET and a sulfonylurea was insufficient to control T2D in the open-label trial AWARD-2 [13]. In that study, after 12 months of treatment, HbA1c was reduced by 1.08% with DU and 0.63% with insulin. There was also a significant difference in the proportion of patients achieving the recommended glycemic targets (53.2% with DU vs. 30.9% with insulin). The statistical superiority of DU over insulin was still present at 18-month study visit, with the advantage of minimal rates of severe hypoglycemic AEs [13]. Consistently, the open-label trial AWARD-5 demonstrated the superior glycemic efficacy of DU 1.5 mg/once weekly over sitagliptin as add-on to MET [12]. Other experimental investigations from the phase-III AWARD clinical trial program, using either a placebo-controlled or an active-comparator design [30,31], along with post-marketing observations [15,16,17,18,19,20], showed benefits of DU treatment on blood glucose in patients with inadequately controlled T2D with prior treatments. Additionally, the retrospective observational studies conducted in Europe and Asia evidenced a mild, but statistically significant, reduction in body weight and other cardiovascular risk factors, such as blood lipids. However, in most cases, efficacy and safety outcomes were assessed after only 6 months of follow-up [15,16,17,19,29], and high heterogeneities in DU regimens and background antidiabetic medications should have been regarded as study limitations. In our cohort of overweight and obese patients with uncontrolled T2D by either MET alone or in selective combination with conventional insulin secretagogues (predominantly a sulfonylurea), the addition of DU 1.5 mg/once weekly significantly improved glycemic control, with a mean 6-month HbA1c reduction of −0.85%, which remained stable for up to 18 months. These results are in remarkable agreement with both clinical trials AWARD-2 and AWARD-5 [12,13], and recent findings of Berra et al., investigating the 12-month effectiveness and safety of DU in a patients’ cohort from Northern Italy [20]. In their multicentric real-world experience, DU significantly improved HbA1c and other metabolic parameters after 6 and 12 months of treatment and increased the percentage of patients reaching target HbA1c levels ≤ 7.0% (from 7.2% at baseline to 55.8% at 12 months) [20]. Similar to our findings, in their cohort, the main reason for DU discontinuation was gastrointestinal intolerance, and there was no patient who dropped out due to severe hypoglycemia [20]. However, despite evidence from preclinical studies suggesting that treatment with GLP-1 RAs might actually protect the kidney from the onset and progression of diabetic microvascular complications [24], a slight reduction in creatinine-based eGFR was noted in our study cohort. Even if statistically superior to that of insulin therapy in patients with moderate kidney failure at 12-month study visit in the open-label trial AWARD 7 [24], compared to other GLP 1-RAs impacting on meaningful kidney-function related outcomes in patients at high cardiovascular risk [32,33], the magnitude of eGFR preservation by DU treatment could seem smaller [34,35]. In absence of dedicated clinical trials, with long-term and comparative follow-up, the effect of DU on the risk of diabetic kidney disease remains equivocal [36], as both DU and LIRA treatments could relate with significant declines in eGFR at 12-month follow-up in real-world scenarios [14].

The burden of T2D on the healthcare system, especially in regions with high prevalence rates, fosters intense clinical and research efforts to determine the most optimal treatment regimen for individual patients with special needs and underlying comorbidities [37]. While searching for factors that would affect clinically relevant glycometabolic outcomes by using correlation and logistic regression analysis, we found that greater baseline HbA1c levels were predictive of better responses to DU, either in terms of HbA1c reduction ≥ 0.5% or body weight loss ≥ 5% at 18-month follow-up. These results are consistent with previous studies with DU and other GLP-1 RA, as baseline HbA1c was the strongest predictor of the magnitude of response in several regression models [17,20]. However, contrary to AWARD clinical trials [38], and previous real-world experiences with LIRA and exenatide from our group and others [8,22,39], we could not demonstrate a greater glycometabolic effect in women. The reasons for these discrepancies are not clear, as a larger exposure to DU and other GLP1-RAs occurs in women [38,40]. Presumably, pharmacokinetic differences are not the sole determinants of sexual dimorphic pharmacological responses, as distinct adherence rates and divergent behaviors toward drugs, other than sex-specific patterns of T2D [41], may influence clinical outcomes following the administration of GLP-1 RAs. Interestingly, for the first time, we could observe that patients with a greater BMI excess were less prone to discontinue DU due to AEs. Among the body weight-lowering drugs approved by the American and European medicine regulatory authorities, LIRA has been put under the spotlight for the intrinsic high risk of drug discontinuation due to AEs (within one year) [42]. Given that the long-term safety and efficacy differ among medications, the ideal approach for body weight loss should be highly individualized, and there is a need for feasible and durable options in obesity pharmacotherapy [42]. Since the completion of our study, higher DU doses (3 mg and 4.5 mg/once weekly) have been approved by the FDA for the management of hyperglycemia in T2D [43]. However, presently, the clinical use of DU is not authorized to improve body weight loss in patients with excess body weight but without T2D, neither at 1.5mg/once weekly nor at expanded doses [43]. 

Although clinical trials should provide the highest level of evidence to guide therapeutic decisions, to date, there is a lack of head-to-head comparisons on the different GLP-1 RA molecules [14]. Furthermore, the results of real-world studies are sometimes contradictory, because of small sample sizes, differences in statistical and laboratory methodologies, or participants’ heterogeneity [44]. As a secondary aim of the study, we compared the long-term effectiveness of DU to that of LIRA, by analyzing two patient cohorts who were highly similar for baseline characteristics, including background medications. After 18 months of continuous treatment, there were no statistically significant differences in the magnitude of body weight loss or HbA1c reduction between the two GLP-1 RAs. Still, a statistically significant larger proportion of patients on DU reached target HbA1c levels than those on LIRA, with the advantage of once-weekly injections. Our findings are coherent with those recently presented by Morieri et al. on multicentric retrospective data gathered from diabetes outpatient clinics in the Veneto region of Northeast Italy, demonstrating that, within/after a mean follow-up of 6 months, HbA1c declined more drastically in patients initiating DU than in those initiating LIRA (on top of MET therapy), despite equivalent body weight loss [29]. Differences in glycemic outcomes between GLP-1 RAs may possibly reflect the inconstant up-titration for LIRA to the highest dose (1.8 mg/once daily) commonly observed in routine practice, often as a means to minimize patients’ risk of gastrointestinal AEs and discontinuation [9,29]. Nonetheless, in a previous study by our group [9], intensification of LIRA dose did not correlate with a greater decrement in HbA1c at 5-year follow-up in patients with T2D and overweight/obesity; therefore, the question about which GLP-1 RAs has higher potency and durability in controlling hyperglycemia under these circumstances remains an open one [29].

A comparatively high genetic homogeneity, like the one found in populations from Calabria, Southern Italy, may allow for a small inter-individual variability in drug responses [9,21,45,46], enhancing the reliability of our findings. However, the present work is not free from limitations, some of which are inherent to the retrospective nature of the study design. Additionally, even though follow-up has been provided for a quite long period of time, the limited sample size of our patient cohort may have inflated—at least to some extent—the statistical soundness of the reported observations. To assess the effects of predictors on long-term effectiveness and safety of DU as add-on therapy to MET or a combination of MET to conventional insulin secretagogues in routine endocrinological practice more precisely, larger studies are warranted.

## 5. Conclusions

Although limited by a retrospective observational design and lack of constant up-titration for LIRA to the highest dose as the active comparator, the findings of this study demonstrate that treatment of T2D with DU 1.5 mg/once weekly on a background of MET or a combination of MET plus conventional insulin secretagogues can lead to durable benefits in relation to glycometabolic control and body weight, with a favorable safety profile, especially in presence of obesity and greater HbA1c impairment.

## Figures and Tables

**Table 1:** Baseline clinical and demographic characteristics of the cohort of overweight or obese type 2 diabetes (T2D) patients eligible for the primary analysis (N = 126) with at least one prescription of dulaglutide 1.5 mg/once weekly as add-on to metformin (MET) or MET plus conventional insulin secretagogues.

Clinical and Demographic Characteristics	N or Means % or (SD)	
Female gender, N	61	48.4%
Age, yr	59.8	(9.4)
T2D duration, yr	8.0	(6.0)
T2D duration >10 yr	41	32.5%
Hypertension, N	97	77.0%
CHD, N	11	8.7%
History of stroke/TIA, N	4	3.2%
Peripheral arterial disease, N	2	1.6%
Overweight (BMI 25–29.9 kg/m^2^), N	46	36.5%
Obesity (BMI ≥ 30 kg/m^2^), N	80	63.5%
Dyslipidemia, N	76	60.3%
Other comorbidities, N	86	68.3%
Microvascular diabetic complications, N	23	18.3%
Diabetic retinopathy, N	9	7.1%
Early-stage DKD, N	8	6.4%
Advanced stage DKD, N	1	0.8%
Diabetic neuropathy, N	9	7.1%
**Background Medications**		
ACE inhibitors, N	34	27.0%
ARB, N	42	33.3%
Calcium blockers, N	15	11.9%
Beta-blockers, N	37	29.4%
Diuretics, N	46	36.5%
Alpha1 blockers, N	7	5.6%
Statin, N	59	46.8%
Ezetimibe, N	6	4.8%
Cardioaspirin, N	23	18.3%
Other medications, N	45	35.7%
MET, N	126	100%
MET plus sulfonylurea, N	18	14.2%
MET plus glinide, N	9	7.1%
Naïve to GLP-1 RAs, N	109	86.5%

Data are presented as N and %, or means (SD), as appropriate. CHD: coronary heart disease; TIA: transient ischemic attack; DKD: diabetic kidney disease; ACE: angiotensin-converting enzyme; ARB: angiotensin receptor blockers; GLP-1 RA: GLP-1 receptor agonist; SD: standard deviations; BMI: body mass index.

**Table 2:** Longitudinal comparisons of safety outcome measures for up to 18 months.

Parameter	Baseline (N = 126)	6 Months (N = 89)	12 Months (N = 77)	18 Months (N = 65)	*p* Value (0 vs. 6 Months)	*p* Value (0 vs. 12 Months)	*p* Value (0 vs. 18 Months)
Creatinine, mg/dL	0.85 (0.19)	0.90 (0.18)	0.85 (0.22)	0.94 (0.19)	0.624	0.906	0.097
eGFR, mL/min	90.1 (25.1)	82.1 (15.0)	91.1 (21.9)	84.1 (20.7)	0.461	1.000	0.049
AST, U/L	26.9 (11.8)	29.6 (19.4)	25.7 (9.0)	29.3 (10.9)	0.819	0.716	0.442
ALT, U/L	31.4 (18.4)	26.0 (11.9)	25.8 (13.2)	24.3 (7.0)	0.041	0.166	0.451

Data are presented as means (SD). Paired data were compared by using the Wilcoxon signed-rank test. Missing values were handled by excluding cases per dependent variable. eGFR: estimated glomerular filtration rate.

**Table 3:** Longitudinal comparisons of efficacy outcome measures for up to 18 months.

Parameter	Baseline (N = 126)	6 Months (N = 89)	12 Months (N = 77)	18 Months (N = 65)	*p* Value (0 vs. 6 Months)	*p* Value (0 vs. 12 Months)	*p* Value (0 vs. 18 Months)
HbA1c, %	7.8 (1.0)	7.0 (1.0)	7.0 (1.2)	6.9 (1.0)	<0.001	<0.001	<0.001
FPG, mg/dL	169.4 (37.9)	140.5 (27.1)	145.0 (38.4)	141.4 (28.3)	<0.001	<0.001	<0.001
Body weight, kg	92.1 (16.9)	89.9 (17.2)	91.8 (17.6)	91.4 (18.5)	<0.001	0.002	0.007
BMI, kg/m^2^	33.2 (5.7)	32.8 (6.0)	33.2 (6.0)	32.8 (6.0)	<0.001	0.003	0.006
Systolic BP, mmHg	129.8 (14.5)	130.9 (14.7)	128.6 (14.6)	131.5 (12.6)	0.407	0.758	0.758
Diastolic BP, mmHg	76.3 (10.5)	77.2 (11.1)	77.3 (10.4)	76.5 (8.2)	0.279	0.084	0.084
Total chol., mg/dL	175.8 (35.6)	164.8 (30.2)	161.7 (32.4)	166.6 (35.5)	0.022	0.005	0.017
LDL-chol., mg/dL	98.9 (31.1)	89.3 (23.7)	87.2 (28.2)	82.8 (28.3)	0.030	0.040	0.065
HDL-chol., mg/dL	47.8 (10.7)	47.4 (11.5)	49.2 (10.7)	49.1 (11.7)	0.786	0.274	0.820
TG, mg/dL	155.9 (110.6)	143.3 (67.0)	140.1 (82.3)	152.9 (61.3)	0.200	0.011	0.763

Data are presented as means (SD). Paired data were compared by using the Wilcoxon signed-rank test. Missing values were handled by excluding cases per dependent variable. LDL: low-density lipoprotein; HDL: high-density lipoprotein; TG: triglycerides; BP: blood pressure; FPG: fasting plasma glucose.

**Table 4:** Logistic regression analyses predicting safety and efficacy outcomes (i.e., drug discontinuation due to adverse events (AEs), HbA1c reduction ≥ 0.5%, body weight loss ≥ 5%) when using dulaglutide 1.5 mg/once weekly as add-on to MET or MET plus insulin secretagogues.

Outcome	Predictor	OR	(95% CI)	*p* Value
Drug discontinuation due to AEs *	Baseline BMI ≥ 30 kg/m^2^	0.211	(0.058–0.771)	0.019
HbA1c reduction ≥ 0.5%	Baseline HbA1c %	2.961	(1.394–6.290)	0.005
Body weight loss ≥ 5%	Baseline HbA1c %	2.571	(1.171–5.644)	0.019

Results are reported as Odds Ratios (OR) with 95% confidence intervals (CI). Each categorical outcome was treated separately. * Age and gender were added as covariates. AEs: adverse events.

**Table 5:** Comparative efficacy between dulaglutide (DU) 1.5 mg/once weekly and liraglutide (LIRA) 1.2–1.8 mg/once daily as add-on to MET or MET plus conventional insulin secretagogues, after 18 months of continuous treatment.

Outcomes	LIRA (N = 54)	DU (N = 54)	*p* Value
Body weight reduction ≥ 5%	15 (27.8%)	13 (24.1%)	0.827
HbA1c reduction ≥ 0.5%	32 (59.3%)	34 (63.0%)	0.987
HbA1c ≤ 7%	23 (42.6%)	35 (64.8%)	0.033

*p* values were calculated by using the Fisher’s exact test.

## Data Availability

Data supporting the reported results are available from the corresponding author upon reasonable request.

## Supplementary Materials

The following are available online at https://www.mdpi.com/2077-0383/10/5/985/s1, Table S1: Longitudinal changes in HbA1c and body weight in patients treated with dulaglutide 1.5 mg/once weekly as add-on to MET or MET plus conventional insulin secretagogues; Table S2: Baseline characteristics of frequency-matched groups (N = 54 both) naïve to GLP-1RA who were treated with dulaglutide (DU) 1.5mg/once weekly or liraglutide (LIRA) 1.2– 1.8mg/once daily as add-on to metformin or metformin plus conventional insulin secretagogues for a minimum of 18 months. Table S3: Longitudinal comparisons between matched samples (N = 54) naïve to GLP-1 RA who were treated with dulaglutide (DU) or liraglutide (LIRA) as add-on to metformin or metformin plus conventional insulin secretagogues for a minimum of 18 months.
