# Supplementary material for: Clinical Effectiveness and Safety of Once-Weekly GLP-1 Receptor Agonist Dulaglutide as Add-On to Metformin or Metformin Plus Insulin Secretagogues in Obesity and Type 2 Diabetes
Source: J Clin Med. 2021 Mar 2;10(5):985. doi: 10.3390/jcm10050985 (PMC7957905; doi:10.3390/jcm10050985)
Supplement: Supplementary file 1 [file jcm-10-00985-s001.pdf]

Supplementary Materials

Table S1. Longitudinal changes in HbA1c and body weight in patients treated with dulaglutide 1.5mg/once weekly as add-on to MET or MET plus conventional insulin secretagogues.

Changes	6 Months (N = 89)	12 Months (N = 77)	18 Months (N = 65)	*p* Value (6 vs. 12 months)	*p* Value (6 vs. 18 months)
HbA1c, %	−0.85 (1.17)	−0.85 (1.25)	−0.75 (1.49)	0.443	0.423
Body weight, %	−2.0 (4.3)	−1.7 (4.7)	−1.3 (4.8)	0.227	0.091
Body weight, kg	−2.8 (9.8)	−1.7 (4.5)	−1.4 (4.5)	0.366	0.087

Data are presented as means (SD). Paired data were compared by using the Wilcoxon signed-rank test. Missing values were handled by excluding cases per dependent variable. Longitudinal changes were calculated with respect to baseline values, as absolute (HbA1c) or relative (body weight) percentage differences. MET: metformin; SD: standard deviations.

Table S2. Baseline characteristics of frequency-matched groups (N = 54 both) naïve to GLP-1 RA who were treated with dulaglutide (DU) 1.5 mg/once weekly or liraglutide (LIRA) 1.2–1.8mg/once daily as add-on to metformin or metformin plus conventional insulin secretagogues for a minimum of 18 months. Matching criteria included age, gender, T2D duration, degree of glycemic impairment, cardiovascular comorbidities, and background medications.

Clinical and demographic characteristics	LIRA (N = 54)		DU (N = 54)		*p* Value
Female gender, N	31	57.4%	31	57.4%	1.000
Age, yr	56.7 (6.3)		58.8 (9.1)		0.134
T2D duration, yr	7.4 (5.0)		7.6 (5.0)		0.824
T2D duration > 10 yr	16	29.6%	15	27.8%	1.000
Hypertension, N	41	75.9%	39	72.2%	0.827
CHD, N	6	11.1%	6	11.1%	1.000
History of stroke/TIA, N	1	1.9 %	2	3.7%	1.000
Peripheral arterial disease, N	1	1.9%	2	3.7%	1.000
Overweight (BMI ≥ 25–29.9 kg/m^2), N	16	29.6%	20	37.0%	0.541
Obesity (BMI ≥ 30 kg/m^2), N	38	70.4%	34	63.0%	0.541
Dyslipidemia, N	36	66.7%	35	64.8%	1.000
Other comorbidities, N	38	70.4%	40	74.1%	0.830
Microvascular diabetic complications, N	15	27.8%	13	24.1%	0.827
Diabetic retinopathy, N	7	13.0%	5	9.3%	0.761
Early stage DKD, N	7	13.0%	5	9.3%	0.761
Advanced stage DKD, N	1	1.9%	1	1.9%	1.000
Diabetic neuropathy, N	2	3.7%	5	9.3%	0.437
Weight, kg	92.9 (17.8)		92.1 (18.8)		0.678
BMI, kg/m^2	33.1 (4.8)		33.1 (6.2)		0.239
Systolic BP, mmHg	132.6 (15.8)		130.2 (14.1)		0.653
Diastolic BP, mmHg	75.7 (10.3)		75.5 (11.3)		0.735
FPG, mg/dL	169.2 (32.9)		173.6 (39.2)		0.770
HbA1c, %	7.9 (0.9)		7.8 (0.9)		0.410
HbA1c ≤ 7%	8	14.8%	8	14.8%	1.000
Creatinine, mg/dL	0.82 (0.22)		0.87 (0.21)		0.236
eGFR, mL/min	98.3 (21.8)		91.6 (26.9)		0.070
Total cholesterol, mg/dL	175.4 (31.4)		188.5 (35.7)		0.158
LDL-cholesterol, mg/dL	96.7 (32.3)		109.6 (32.1)		0.209
HDL-cholesterol, mg/dL	45.2 (7.7)		48.7 (11.5)		0.070
Triglycerides, mg/dL	166.3 (64.9)		168.1 (144.9)		0.127
AST, U/L	25.4 (10.2)		24.5 (9.2)		0.880
ALT, U/L	30.6 (15.6)		29.8 (14.8)		0.915
**Background Medications**					
ACE inhibitors, N	17	31.5%	15	27.8%	0.833
ARB, N	18	33.3%	16	29.6%	0.835
Calcium blockers, N	13	24.1%	7	13.0%	0.214
Beta blockers, N	15	27.8%	15	27.8%	1.000
Diuretics, N	24	44.4%	17	31.5%	0.231
Alpha1 blockers, N	3	5.6%	3	5.6%	1.000
Statin, N	27	50.0%	28	51.9%	1.000
Ezetimibe, N	4	7.4%	3	5.6%	1.000
Cardioaspirin, N	12	22.2%	13	24.1%	1.000
MET, N	54	100.0%	54	100.0%	1.000
MET + sulfonylurea, N	10	18.5%	7	13.0%	0.598
MET + glinide, N	3	5.6%	4	7.4%	1.000

Data are presented as N and % or means (SD). *p* values were calculated by using the Fisher's exact test or the Mann-Whitney U test, as appropriate. T2D: type 2 diabetes; CHD: coronary heart disease; TIA: transient ischemic attack; DKD: diabetic kidney disease; BMI: body mass index; BP: blood pressure; FPG: fasting plasma glucose; eGFR: estimated glomerular filtration rate; AST: aspartate aminotransferase; ALT: alanine aminotransferase; ACE: angiotensin-converting enzyme; ARB: angiotensin receptor blockers.

Table S3. Longitudinal comparisons between matched samples (N = 54) naïve to GLP-1 RA who were treated with dulaglutide (DU) or liraglutide (LIRA) as add-on to metformin or metformin plus conventional insulin secretagogues for a minimum of 18 months.

	**6 Months**		**12 Months**		**18 Months**		***p* Value**	***p* Value**	***p* Value**
	**LIRA**	**DU**	**LIRA**	**DU**	**LIRA**	**DU**	**(6 Months)**	**(12 Months)**	**(18 Months)**
HbA1c, %	7.2 (0.9)	6.9 (1.0)	7.1 (0.9)	6.9 (1.0)	7.2 (1.0)	6.9 (0.9)	0.061	0.224	0.088
FPG, mg/dL	145.6 (33.1)	136.2 (24.7)	142.2 (37.8)	139.3 (29.4)	146.5 (27.2)	139.6 (25.5)	0.249	0.922	0.183
Body weight, kg	89.1 (15.9)	89.0 (19.0)	90.0 (17.5)	91.4. (21.0)	90.1 (17.7)	90.7 (18.6)	0.811	0.897	0.955
BMI, kg/m^2	31.8 (3.6)	32.0 (6.2)	31.7 (3.7)	33.6 (9.4)	32.0 (3.9)	32.6 (6.0)	0.299	0.920	0.734
Systolic BP, mmHg	130.2 (12.2)	129.3 (16.2)	132.4 (13.7)	128.8 (11.1)	132.3 (18.4)	130.7 (11.0)	0.544	0.344	0.750
Diastolic BP, mmHg	77.5 (11.3)	76.0 (11.4)	75.1 (7.8)	76.5 (8.2)	76.5 (8.8)	75.9 (8.7)	0.634	0.416	0.985
Creatinine, mg/dL	0.81 (0.29)	0.96 (0.10)	0.85 (0.24)	0.92 (0.27)	0.84 (0.22)	0.95 (0.20)	0.007	0.380	0.031
eGFR, mL/min	96.5 (29.2)	78.1 (9.0)	92.0 (21.5)	86.9 (20.9)	95.8 (18.3)	82.2 (20.9)	0.084	0.558	0.019
Total chol., mg/dL	163.4 (21.5)	167.5 (31.5)	165.4 (28.0)	168.8 (32.8)	166.6 (31.0)	169.8 (37.1)	0.657	0.626	0.602
LDL-chol., mg/dL	87.3 (23.0)	97.6 (24.2)	86.9 (25.6)	93.6 (30.1)	89.3 (26.0)	83.9 (29.9)	0.283	0.574	0.435
HDL-chol., mg/dL	47.4 (11.6)	44.9 (13.4)	47.3 (12.3)	48.9 (9.8)	48.7 (48.3)	48.3 (12.7)	0.251	0.320	0.808
Triglycerides, mg/dL	146.0 (83.6)	147.9 (65.0)	153.7 (86.8)	130.1 (49.8)	146.6 (69.7)	157.8 (63.2)	0.593	0.599	0.354
AST, U/L	27.1 (10.1)	26.7 (8.5)	27.8 (8.4)	25.5 (8.9)	35.2 (16.2)	29.8 (11.5)	0.869	0.333	0.198
ALT, U/L	32.9 (13.5)	24.7 (7.6)	31.6 (13.5)	24.0 (10.5)	35.7 (20.7)	24.9 (7.2)	0.043	0.077	0.032
				**Changes**					
HbA1c, %	−0.6 (1.1)	−0.9 (1.3)	−0.8 (1.0)	−1.0 (1.3)	−0.76 (1.1)	−0.82 (1.5)	0.279	0.314	0.714
Body weight, %	−3.2 (4.5)	−3.0 (4.2)	−3.4 (3.7)	−2.1 (5.4)	−3.0 (3.7)	−1.7 (4.5)	0.800	0.249	0.160
Body weight, kg	−3.0 (4.3)	−2.8 (4.2)	−3.2 (3.5)	−1.9 (5.6)	−2.8 (3.4)	−1.6 (4.2)	0.781	0.223	0.163

Data are presented as means (SD). *p* values were calculated by using the Mann-Whitney U test.
